# Myocardial protection during surgery for infective endocarditis: retrospective, single center, risk-adjusted study

**DOI:** 10.1186/s13019-026-04246-y

**Published:** 2026-05-05

**Authors:** Murat Mukharyamov, Tulio Caldonazo, Philine Fleckenstein, Sebastian Freiburger, Hristo Kirov, Mathias Pletz, Stefan Hagel, Jürgen Bogoviku, Sandesh Dinesh, Stefan Glöckner, Micha Banz, Mahmoud Diab, Torsten Doenst, the IKARUS study group, Mathias W. Pletz, Bettina Löffler, Christian Schulze, Regine Heller

**Affiliations:** 1 Department of Cardiothoracic Surgery, Jena University Hospital, Friedrich Schiller University of Jena, Am Klinikum 1, 07747 Jena, Germany; 2 Institute of Infectious Diseases and Infection Control, Jena University Hospital, Friedrich Schiller University of Jena, Jena, Germany; 3 Department of Internal Medicine I: Cardiology, Angiology, Intensive Care Medicine, Jena University Hospital, Friedrich Schiller University of Jena, Jena, Germany; 4 Institute of Medical Microbiology, Jena University Hospital, Friedrich Schiller University of Jena, Jena, Germany; 5 Department of Internal Medicine IV: Gastroenterology, Hepatology, Infectious Diseases, Interventional Endoscopy), Jena University Hospital, Friedrich Schiller University of Jena, Jena, Germany; 6 Department of Cardiac Surgery, Helios University Hospital Wuppertal, University of Witten/Herdecke, Wuppertal, Germany

**Keywords:** Infective endocarditis, Cardioplegia, Myocardial protection

## Abstract

**Objectives:**

Current evidence does not support superiority of one cardioplegia type over another, but stems from low-risk populations. Therefore, we compared outcomes of multimorbid, high-risk infective endocarditis (IE) patients receiving Custodiol^®^crystalloid or Calafiore blood cardioplegia during cardiac surgery.

**Methods:**

We retrospectively analyzed 553 patients (mean EuroScore II 22.7 ± 21.1) who underwent surgery for IE between 2009 and 2023 and received either cold crystalloid (Custodiol^®^, *n* = 335) or warm blood (Calafiore, *n* = 218) cardioplegia. The primary endpoint was 1-year mortality. Secondary endpoints included 30-day mortality, postoperative stroke, and new-onset dialysis. Propensity score matching (1:1, 14 covariates) resulted in 175 matched pairs. Statistical analysis included nonparametric and exact tests.

**Results:**

In the overall cohort, patients receiving Custodiol^®^ were higher risk and had higher mortality and morbidity. After matching, there was no significant difference in 1-year mortality between patients receiving Custodiol^®^ and Calafiore (37.1% vs. 28.6%, *p* = 0.09). 30-day mortality trended to be lower in the Calafiore group without reaching statistical significance (22.9% vs. 14.9%, *p* = 0.057). However, stroke was less frequent (4.6% vs. 10.9%, *p* = 0.029), ICU stay was shorter (3[1–8] vs. 6[3–12.5] days, *p* < 0.001) and postoperative dialysis was numerically less common (13.7 vs. 20.6%, *p* = 0.091). These differences were most evident in procedures with shorter cross-clamp times, such as isolated mitral or aortic valve surgery, where mortality and recovery parameters consistently favored Calafiore.

**Conclusions:**

In high-risk endocarditis patients warm blood cardioplegia may be superior to cold crystalloid, although differences did not reach statistical significance. However, propensity matching may not have accounted for all differences, which warrants further discussion and investigation.

**Supplementary Information:**

The online version contains supplementary material available at 10.1186/s13019-026-04246-y.

## Introduction

Infective endocarditis (IE) remains a clinical condition associated with high mortality and substantial risk of perioperative complications. Surgical treatment is a key component of the management strategy for many IE patients [1]. Current IE guidelines provide detailed recommendations on diagnosis, risk stratification and the organization of multidisciplinary endocarditis teams. Yet, they do not offer specific recommendations regarding the choice of cardioplegic solution or myocardial protection strategy in this population, leaving the decision to the surgical team and the operative context [1]. In parallel, the updated EACTS/EACTAIC/EBCP perfusion guidelines focus on the principles of perfusion (temperature, delivery routes and regimens, hemoconcentration/hemodilution, adjunctive measures), emphasizing the need for a standardized but individualized approach; at the same time, no “best” cardioplegic solution is designated as a universal standard [2]. This uncertainty is also reflected in real-world practice, with a global survey revealing substantial variability in cardioplegic strategies across centers and regions [3].

In our recent review on myocardial protection [4], we demonstrated that the fundamental approaches to cardioplegia have remained largely unchanged over the past decades, whereas the patient profile has markedly shifted towards older age and higher comorbidity. While there is no convincing evidence of the superiority of any solution for “hard” outcomes, most of these data were obtained in low-risk populations. At the same time, other factors such as systemic (extra-cardiac) effects of cardioplegia, incl. vasoplegia or the affection of renal or cerebral function, may be of particular relevance in high-risk groups.

Patients with IE may reflect such a high risk group where anatomic and technical factors (aortic regurgitation, root destruction, multiple valve involvement) often require individualized approaches to myocardial protection (e.g., combined or retrograde delivery for variable aortic cross-clamp times). In such situations, potential differences between solutions and regimens (e.g., in vasoplegic profile, degree of hemodilution or frequency of re-dosing) may influence clinical outcomes not only through cardio-specific protection but also via systemic effects — a gap highlighted in our contemporary review and not addressed in current guidelines [4].

Based on these considerations, the aim of the present study was to assess whether the type of cardioplegia influences the outcomes of IE surgery in a real-world, high-risk cohort, focusing not only on mortality but also on clinically relevant secondary outcomes (stroke, renal dysfunction/dialysis, ICU and hospital length of stay), as well as potential differences across surgical subgroups.

## Methods

### Study design and patient population

We conducted a retrospective single-center cohort study including all adult patients who underwent surgery for infective endocarditis (IE) at Jena University Hospital between January 2009 and December 2023. The study was approved by the Ethics Committee of Jena University Hospital. Informed consent was waived due to the retrospective design. The study was conducted in compliance with the Declaration of Helsinki.

The diagnosis of IE was established according to the Duke criteria 2000 and modified version after 2023 [5, 6], integrating clinical presentation, echocardiographic findings, and microbiological evidence from blood cultures and/or valve specimens. Both native and prosthetic valve IE, as well as isolated and combined valve procedures, were eligible. Patients without available cardioplegia data or undergoing surgery without cardiopulmonary bypass (CPB) were excluded.

Myocardial protection strategies.

Two cardioplegia protocols were applied during the study period. First, cold crystalloid cardioplegia (Custodiol^®^ HTK, Köhler Chemie GmbH, Alsbach-Hähnlein, Germany) was administered as a single-dose antegrade infusion (2000 ml) at 4–8 °C, with repeat dosing (500–700 ml) in cases of prolonged aortic cross-clamp time exceeding 90 min. Second, intermittent antegrade warm blood cardioplegia was delivered according to the protocol described by Calafiore [7], at 34–37 C at 20-minute intervals. The choice of strategy was at the surgeon’s discretion. Retrograde or combined delivery was used in cases with severe aortic regurgitation, root destruction, or complex reoperations according to surgeon preference. According to the institutional standard protocol, cardiopulmonary bypass was performed under normothermic conditions, regardless of the cardioplegic strategy applied.

Data collection and variable definitions.

All study data were derived from the institutional electronic patient charts. Demographic information included age, sex, and body mass index. Clinical variables comprised relevant comorbidities, preoperative functional status, left ventricular ejection fraction, renal function, and preoperative risk assessment using the EuroSCORE II. Endocarditis-related characteristics included valve localization, vegetation size determined by echocardiography or intraoperative inspection, history of recurrent IE, and microbiological results from blood and/or valve examination. Intraoperative variables included procedure type, cardiopulmonary bypass time, aortic cross-clamp time, total operative time, concomitant procedures, transfusion requirements, and use of inotropes or vasopressors.

The primary endpoint was all-cause mortality at 1 year. Secondary endpoints were 30-day mortality, postoperative stroke, new-onset dialysis, intensive care unit (ICU) length of stay, and total postoperative hospital length of stay. A postoperative stroke was defined as a new focal neurological deficit of sudden onset, persisting for at least 24 h, and confirmed by computed tomography or magnetic resonance imaging. New-onset dialysis was defined as initiation of renal replacement therapy in the postoperative period in patients without preoperative dialysis dependence.

Statistical methods.

Statistical analysis and data visualization were conducted using R 4.5.0 statistical computing environment (R Foundation for Statistical Computing, Vienna, Austria). Descriptive statistics for categorical variables are presented with absolute and relative frequencies. Quantitative variables with symmetrical distributions are described using mean (standard deviation), and variables with skewed distributions are presented with median (1st; 3rd quartiles). Propensity score matching (PSM) was performed using the MatchIt 4.7.1 package. To assess covariate balance of potential confounders, standardized mean difference was used. Logistic regression was employed for PSM, with untransformed covariates included as predictors. The nearest neighbor method without caliper was used for 1 to 1 PSM. For comparing full cohort groups regarding quantitative variables, the Brunner-Munzel test was used, while for comparing groups in terms of binary and categorical variables, the exact Fisher test with mid-p correction and exact version of Pearson’s chi-squared test are applied. For comparing matched groups regarding quantitative variables, the Wilcoxon signed-rank test was used. Relative risks were extimated using log-binomial generalized estimating equations (GEE) models. For survival analysis were used Kaplan-Meier estimator, univarialbe Cox proportional hazards model with likelihood ratio test. Clustered Cox regression was used for survival analisys after PSM.

## Results

Baseline demographic and clinical data are summarized in Table 1. Patients in the Calafiore group less often had previous cardiac surgery (17.4% vs. 28.7%, *p* = 0.002), but more frequently presented with NYHA class IV symptoms (35.3% vs. 16.5%, *p* < 0.001) and LVEF < 50% (46.3% vs. 33.1%, *p* = 0.002). Other baseline variables, including sex, renal function, and major comorbidities, did not differ between groups.

**Table 1:** Baseline demographic and clinical data

Characteristics	Total *n* = 553	Custodiol *n* = 335	Calafiore *n* = 218	*p*
Sex (female) (%)	26.6%	26.6%	26.6%	0.99
Age [years] median (q1;q3)	65 (55; 73)	67 (58; 74)	63 (51.2; 71.8)	< 0.001
BMI [kg/m²] median (q1;q3)	26.6 (24.2; 29.8)	26.9 (24.2; 30.8)	26.2 (23.7; 29.3)	0.04
GFR [ml/min] median (q1;q3)	70.8 (44; 94.6)	70.8 (46.4; 92.6)	72.9 (40.4; 101)	0.436
Creatinine [µmol/L] median (q1;q3)	93 (70; 132.8)	92 (70; 128)	97 (74; 143)	0.151
EuroSCORE II mean (SD)	22.7 (21.1)	22.8 (20.9)	22.6 (21.4)	0.789
Pulmonary disease (%)	8.3	9	7.3	0.51
Extracardiac arteriopathy (%)	9.2	9	9.6	0.784
Neurological dysfunction (%)	19.7	20.3	18.8	0.671
Previous cardiac surgery (%)	24.2	28.7	17.4	0.002
Diabetes on insulin (%)	16.6	16.7	16.5	0.955
Critical pre-op state (%)	53.8	53.3	62.5	0.642
Recent MI (%)	4.7	5.7	3.2	0.187
NYHA class IV (%)	23.9	16.5	35.3	< 0.001
LVEF %, median (q1;q3)	55 (50; 60)	56 (50; 63)	54.5 (45; 60)	0.002
LVEF < 50% (%)	38.3	33.1	46.3	0.002

Endocarditis-specific characteristics are shown in Table 2. Aortic valve IE was more common in patients receiving Calafiore (46.4% vs. 27.1%, *p* < 0.001), while mitral (29.9% vs. 21.4%, *p* < 0.001) and multivalve involvement (42.7% vs. 32.3%, *p* < 0.001) were more frequent in patients with Custodiol^®^. Median vegetation size was 12.0 to 13.0 mm. Surgery for recurrent IE was less frequent in the Calafiore group (7.7% vs. 30.2%, *p* < 0.001). Microbiological findings, including Staphylococcus aureus, Enterococcus spp., Candida spp., Streptococcus spp., HACEK, Enterobacteriaceae, and Pseudomonas aeruginosa, showed no relevant differences.

**Table 2:** Endocarditis Details

Characteristics	Total *(n = 553)*	Custodiol *(n = 335)*	Calafiore *(n = 218)*	*p*
**IE localization (%)**				< 0.001
Mitral	26.7	29.9	21.4	
Aortic	34.4	27.1	46.4	
More or equal to 2 valves	38.7	42.7	32.3	
Tricuspid	0.2	0.3	0	
Vegetation size (mm) median (q1;q3)	12 (2; 25)	13 (2; 27)	12 (2; 19.5)	0.002
Vegetation > 15 mm (%)	45.3	48.2	40.6	0.077
Recurrent endocarditis	25.5)	30.2	7.7	< 0.001
**Original Microbiology (%)**				
Staphylococcus aureus	26.5	28.3	23.6	0.223
Enterococcus spp.	11.6	11.1	2.3	0.647
Coagulase-negative staphylococci	11.7	11.6	11.9	0.925
Candida spp.	5.7	6.6	4.1	0.205
Streptococcus spp.	9	9.7	7.8	0.437
HACEK group	4 (0.7%)	1 (0.3%)	1.4	0.174
Enterobacteriaceae	0.3	0	0.9	0.142
Pseudomonas aeruginosa	0.2	0	0.5	0.378

Table 3 shows operative data. Calafiore was more often used in aortic valve surgery procedure (46.8% vs. 29.3%, *p* < 0.001) and less in mitral (21.6% vs. 32.2%, *p* = 0.006) and Bentall/UFO procedures (6.9% vs. 12.8%, *p* = 0.024). Minimally invasive access was more frequent in the Custodiol^®^ group (12.8% vs. 5.0%, *p* = 0.002). The retrograde cardioplegia was used predominantly in the warm blood cardioplegia group. It was administered in more than half of the patients in the Calafiore group (57%), whereas it was applied only in isolated cases in the Custodiol group (1,8%). CPB, cross-clamp, and total operative times did not differ. Use of fresh frozen plasma and milrinone was more frequent in the warm blood cardioplegia group, whereas other transfusions and vasopressor use were similar (Table 3).

**Table 3:** Operative Data

Characteristics	Total *(n = 553)*	Custodiol *(n = 335)*	Calafiore *(n = 218)*	*p*
**Procedure**				< 0.001
Aortic valve surgery	200 (36.2%)	98 (29.3%)	102/218 (46.8%)	< 0.001
Mitral valve surgery	155 (28%)	108 (32.2%)	47/218 (21.6%)	0.006
Double valve surgery	140 (25.3%)	86 (25.7%)	54/218 (24.8%)	0.815
Bentall / UFO	58 (10.5%)	43(12.8%)	15/218 (6.9%)	0.024
Concomitant CABG	79 (14.3%)	46 (13.7%)	33/218 (15.1%)	0.643
MIC	54 (9.8%)	43 (12.8%)	11/218 (5%)	0.002
**Time parameters**				
Bypass time (min)	118 (90; 163.5)	119 (93; 166)	113 (84.5; 161)	0.182
Cross-clamp time (min)	77 (58; 107)	77 (59; 107)	77 (54.8; 104.2)	0.638
Total OR time (min)	199 (159; 259)	199 (158; 268)	198.5 (160; 246.5)	0.895
**Coagulation and transfusion**				
Red blood cells	259 (44.6%)	152 (42.1%)	107 (48.6%)	0.126
Fresh frozen plasma	89 (15.4%)	26 (7.2%)	63 (28.8%)	< 0.001
Platelets	59 (10.2%)	30 (8.3%)	29 (13.3%)	0.059
Fibrinogen	16 (2.8%)	13 (3.6%)	3 (1.4%)	0.113
PPSB	20 (3.4%)	15 (4.2%)	5 (2.3%)	0.237
Factor XIII	11 (1.9%)	9 (2.5%)	2 (0.9%)	0.189
**Vasopressors and Inotropic support**				
Noradrenaline	461 (97.9%)	352 (98.1%)	215 (97.3%)	0.632
Adrenaline	136 (28.9%)	100 (27.9%)	70 (32.1%)	0.384
Milrinone	129 (27.4%)	89 (24.8%)	79 (35.7%)	0.027

**Table 4:** Outcomes (after PSM)

Characteristics	Custodiol *(n = 175)*	Calafiore *(n = 175)*	*p*
**Overall**			
30-day mortality	40/175 (22.9%)	26/175 (14.9%)	0.057
1-year mortality	65/175 (37.1%)	50/175 (28.6%)	0.09
Postoperative stroke	19/175 (10.9%)	8/175 (4.6%)	0.029
Postoperative dialysis	36/175 (20.6%)	24/175 (13.7%)	0.091
Hospital stay [days (q1;q3)]	20 (13; 30)	15 (11; 26)	0.011
ICU stay [days (q1;q3) ]	6 (3; 12.5)	3 (1; 8)	< 0.001
**Aortic valve surgery**			
30-day mortality	13/87 (14.9%)	10/87 (11.5%)	0.513
1-year mortality	22/87 (25.3%)	20/87 (23%)	0.727
Postoperative stroke	6/87 (6.9%)	2/87 (2.3%)	0.171
Postoperative dialysis	9/87 (10.3%)	10/87 (11.5%)	0.814
Hospital stay [median days (q1;q3)]	20 (14; 27.5)	15 (11; 29)	0.09
ICU stay stay [median days (q1;q3)]	6 (3; 10.5)	2 (1; 6)	< 0.001
**Mitral valve surgery**			
30-day mortality	10/34 (29.4%)	4/34 (11.8%)	0.083
1-year mortality	16/34 (47.1%)	7/34 (20.6%)	0.024
Postoperative stroke	5/34 (14.7%)	3/34 (8.8%)	0.484
Postoperative dialysis	6/34 (17.6%)	4/34 (11.8%)	0.519
Hospital stay stay [median days (q1;q3)]	19 (10.2; 30.5)	15.5 (10.8; 21.8)	0.217
ICU stay stay [median days (q1;q3)]	6 (3; 12)	2 (0; 5.8)	0.005
**Double valve surgery**			
30-day mortality	13/43 (30.2%)	9/43 (20.9%)	0.338
1-year mortality	21/43 (48.8%)	19/43 (44.2%)	0.673
Postoperative stroke	5/43 (11.6%)	2/43 (4.7%)	0.271
Postoperative dialysis	19/43 (44.2%)	6/43 (14%)	0.002
Hospital stay stay [median days (q1;q3)]	22 (12.5; 34	15 (10.2; 28)	0.201
ICU stay stay [median days (q1;q3)]	10 (4; 13)	4 (2; 11.5)	0.098
**Bentall / UFO**			
30-day mortality	4/11 (36.4%)	3/11 (27.3%)	0.681
1-year mortality	6/11 (54.5%)	4/11 (36.4%)	0.434
Postoperative stroke	3/11 (27.3%)	1/11 (9.1%)	0.338
Postoperative dialysis	2/11 (18.2%)	4/11 (36.4%)	0.392
Hospital stay stay [median days (q1;q3)]	21 (8; 35.5)	15 (8.5; 24)	0.558
ICU stay stay [median days (q1;q3)]	14 (5; 21)	4 (1.5; 8)	0.055

In the overall cohort, no statistically significant differences in 1-year mortality were observed between groups across any surgical category (Table 4). The causes of 30-day mortality in the Custodiol and Calafiore groups were as follows: multiorgan failure (62.4% vs. 64.7%), cardiac therapy limitation (23.5% vs. 8.8%), cerebral events (9.4% vs. 8.8%), sudden cardiac death (2.4% vs. 2.9%), refractory heart failure (1.2% vs. 2.9%), septic shock (1.2% vs. 0%), and unknown causes (0% vs. 11.8%). In aortic valve surgery, patients in the Calafiore group had a shorter ICU (median 2 vs. 5 days, *p* < 0.001) and hospital stay (15 vs. 19.5 days, *p* = 0.026); other endpoints, including 30-day mortality, stroke, and dialysis, did not differ. In mitral valve surgery, ICU stay was markedly shorter in patients with Calafiore cardioplegia (1 vs. 6 days, *p* < 0.001), with no significant differences in survival or other complications. For double valve procedures, dialysis was more frequent (32.6% vs. 14.8%, *p* = 0.019) and ICU stay was longer (7 vs. 3.5 days, *p* = 0.043) in the Custodiol^®^ group, while mortality and stroke rates were comparable. In Bentall/UFO operations, outcomes were similar, with a non-significant trend toward shorter ICU stay with Calafiore (4 vs. 8 days, *p* = 0.148). We performed an additional subgroup analysis of patients with prolonged aortic cross-clamp time (> 90 min). This subgroup included 194 of 553 patients (35.1%): 112/335 (33.4%) in the Custodiol group and 82/218 (37.6%) in the Calafiore group. In this subgroup, 30-day mortality was 35.9% in the Custodiol group versus 2.2% in the Calafiore group (*p* = 0.059), and 1-year mortality was 47.3% versus 43.4% (*p* = 0.576), respectively.

After propensity score matching using 14 preoperative variables (age, body mass index, NYHA class IV, left ventricular ejection fraction < 50%, recent myocardial infarction < 90 days, diabetes on insulin, pulmonary disease, extracardiac arteriopathy, neurological dysfunction, glomerular filtration rate, previous cardiac surgery, and cross-clamp time), baseline characteristics were well balanced between groups.

In aortic valve surgery, ICU stay was again shorter with Calafiore (2 vs. 6 days, *p* < 0.001) with no significant differences in survival, stroke, or dialysis. In mitral valve surgery, 1-year mortality was lower with Calafiore (20.6% vs. 47.1%, *p* = 0.024) and ICU stay shorter (2 vs. 6 days, *p* = 0.005); other outcomes did not differ. For double valve procedures, dialysis remained more frequent with Custodiol^®^ (44.2% vs. 14.0%, *p* = 0.002), with similar mortality and stroke rates. In Bentall/UFO procedures, there were no significant differences in any endpoint.

## Discussion

We demonstrate in this analysis that in high-risk endocarditis patients, warm blood cardioplegia may be superior to cold crystalloid, although differences did not reach statistical significance. However, propensity matching may not have accounted for all differences, which warrants further discussion and investigation.

We used propensity score matching with 13 preoperative variables achieving a good balance with standardized mean differences below 0.1 for all covariates. In this adjusted cohort, 1-year mortality did not differ significantly between the two cardioplegic strategies. This finding aligns with previous reports in mixed cardiac surgical populations, where no clear survival benefit could be attributed to either crystalloid or blood [8], cold or warm [9] cardioplegia once baseline risk was accounted for. However, numerically one year mortality was lower with Calafiore.

Secondary outcomes also revealed consistent trends suggesting systemic differences between the regimens. Patients receiving warm blood cardioplegia had faster postoperative recovery, shorter ICU stay (2 vs. 6 days, *p* = 0.001), fewer postoperative strokes (5.0% vs. 9.6%), and less frequent dialysis in double-valve procedures (14.8% vs. 32.6%). These differences were most pronounced in operations with shorter aortic cross-clamp times, particularly isolated aortic and mitral valve procedures.

The disappearance of these differences with increasing surgical complexity and cross-clamp time suggests that the observed effects are unlikely to stem from myocardial protection itself, because shorter cross-clamp times require less perfect protection for sufficient endogenous ischemia tolerance. A possible and plausible explanation may therefore lie in the systemic effects of the cardioplegic solution. Custodiol is administered as a single large-volume crystalloid dose and, when allowed to enter the systemic circulation, may induce vasoplegia or contribute to hemodilution. Previous reports demonstrated that active evacuation of Custodiol^®^ from the coronary sinus can attenuate such effects [10], but this maneuver was rarely performed in our series. In shorter and less complex operations, Calafiore cardioplegia is administered in comparatively smaller volumes, and the systemic impact of its components may therefore play a less prominent role. In infective endocarditis, where septic vasoplegia and endothelial dysfunction are common, even modest differences in intravascular volume or electrolyte composition may potentially influence postoperative stability and recovery.

Placed within this physiological context, these findings gain further clarity when contrasted with outcomes reported in major European multicentre registries. Our outcomes are comparable to those reported by the major contemporary registries of surgically treated infective endocarditis, including the EURO-ENDO registry [11] and the German multicentre CAMPAIGN [12] study. Notably, the operative risk in these cohorts was substantially lower—for example, the CAMPAIGN registry reported a median EuroSCORE II of 10–12%, whereas in our study the mean EuroSCORE II was 22.7. Despite this markedly higher risk profile, early mortality in our cohort remained within the range observed in these large registries. Thus, our results can be considered externally valid and potentially generalizable to other centers.

Taken together, these findings suggest that systemic factors may influence postoperative morbidity and recovery in high-risk populations. The potential advantage of warm blood cardioplegia in shorter, less complex procedures may therefore reflect attenuation of systemic effects rather than differences in myocardial preservation. Prospective randomized studies are warranted to confirm these hypotheses and to define the optimal myocardial protection strategy for infective endocarditis surgery. In the meantime, it may be advisable to evacuate Custodiuol solutions from the coronary sinus in infective endocarditis patients, the way custodial usage was initially described [13].

### Limitations

This was a retrospective single-center analysis, with potential for residual confounding despite propensity matching. Some subgroups, particularly Bentall/UFO, were small and underpowered for detecting modest effects.

## Electronic Supplementary Material

Supplementary Material 1

## Data Availability

The datasets generated and/or analyzed during the current study are available from the corresponding author on reasonable request.
