# Supplementary material for: Myocardial protection during surgery for infective endocarditis: retrospective, single center, risk-adjusted study
Source: J Cardiothorac Surg. 2026 May 5;21:216. doi: 10.1186/s13019-026-04246-y (PMC13151226; doi:10.1186/s13019-026-04246-y)

**Supplementary Material**

**Supplementary figures legends**

**Supplementary Figure 1** - Kaplan–Meier Analysis of Overall Survival in the Full Cohort. Curves depicting overall survival in patients undergoing surgery for infective endocarditis receiving Custodiol crystalloid cardioplegia or Calafiore warm blood cardioplegia. Follow-up ranged from 0 days to 164.days (IQR: 0.7–57.6 days). The difference was not statistically significant (hazard ratio [HR] 0.88, 95% confidence interval [CI] 0.69–1.13; p = 0.313), as determined by a univariable Cox proportional hazards model using the likelihood ratio test.

**Supplementary Figure 2** - Kaplan–Meier curves illustrating overall survival in propensity score–matched patients undergoing surgery for infective endocarditis who received Custodiol crystalloid cardioplegia or Calafiore warm blood cardioplegia. Follow-up ranged from 0 days to 160.9 days (IQR: 0.8–59.2 days). The difference was not statistically significant (hazard ratio [HR] 0.88, 95% confidence interval [CI] 0.65–1.20; p = 0.43 as determined by clustered Cox proportional hazards regression.

**Supplementary Figure 3** - Love Plot Demonstrating Covariate Balance Before and After Propensity Score Matching. Standardized mean differences (SMDs) of baseline covariates between the Custodiol and Calafiore groups before and after propensity score matching (PSM). The vertical dashed line represents the predefined threshold for acceptable balance (SMD < 0.1).

**Supplementary Figure 1**


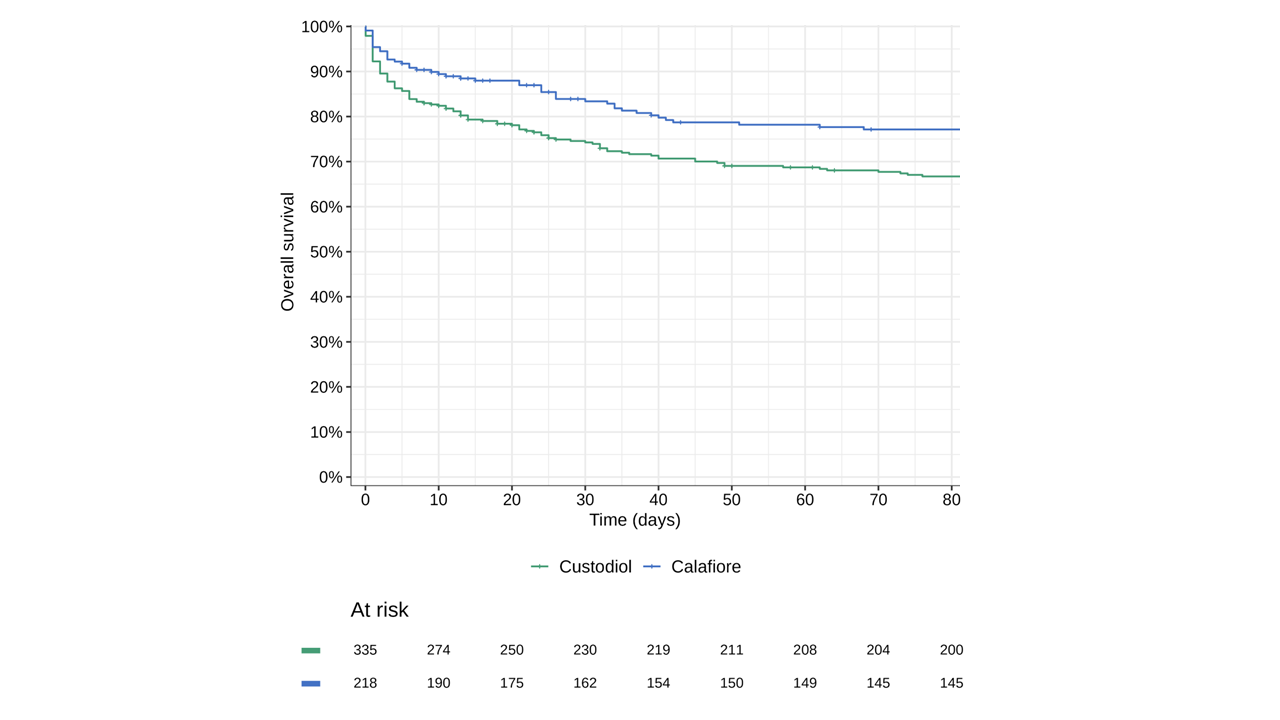


**Supplementary Figure 2**

**
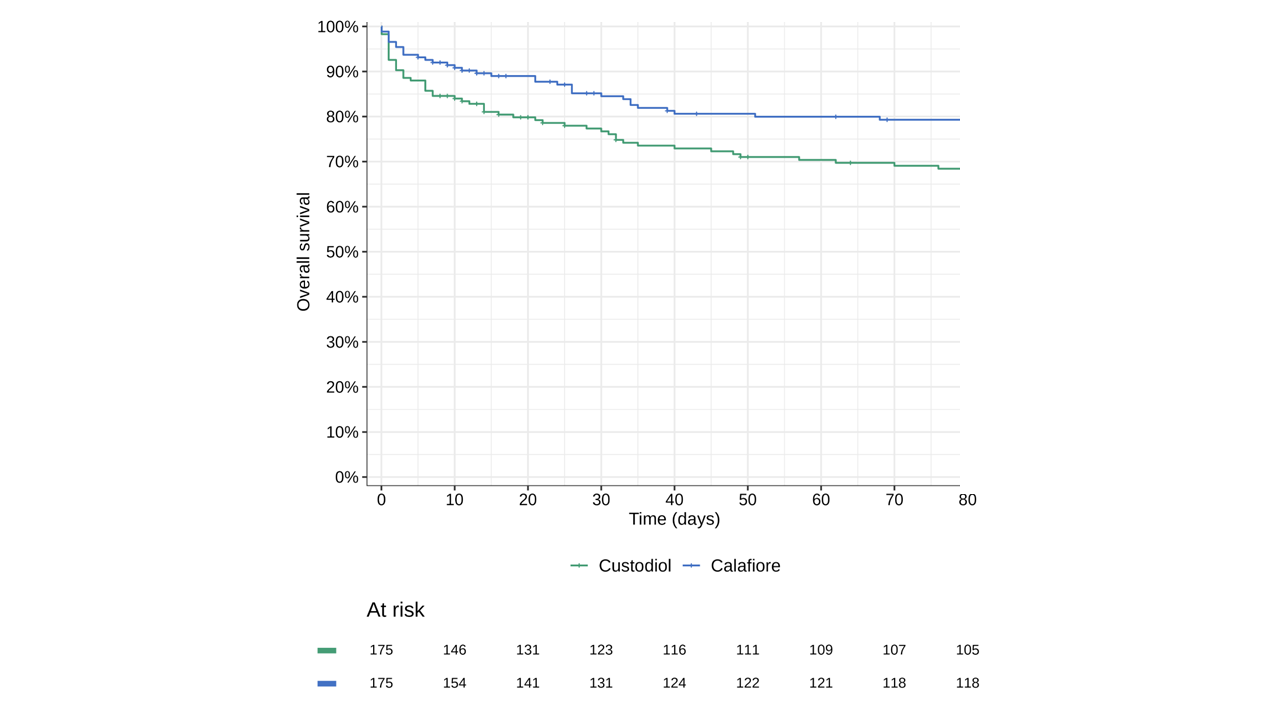
**

**Supplementary Figure 3**


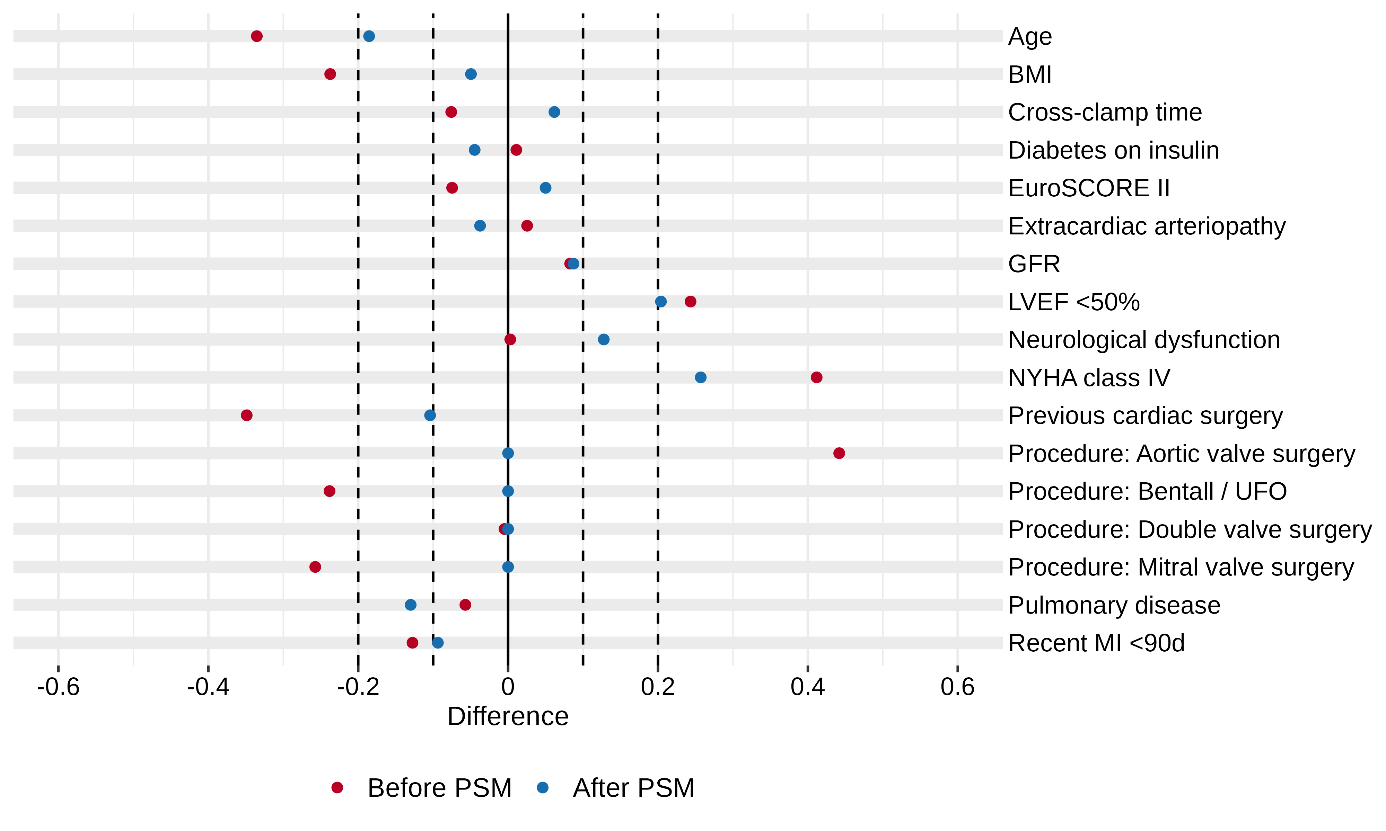

Supplement: Supplementary file 1 — Supplementary Material 1 [file 13019_2026_4246_MOESM1_ESM.docx]
